# Promiscuity‐Guided Enzyme Evolution via Substrate Multiplexed Screening

**DOI:** 10.1002/anie.202600007

**Published:** 2026-05-11

**Authors:** Holly A. Weilbaker, Meghan E. Campbell, Peyton M. Higgins, Andrew R. Buller

**Affiliations:** ^1^ Department of Chemistry University of Wisconsin‐Madison Madison Wisconsin USA; ^2^ Department of Chemistry Smith College Northampton Massachusetts USA; ^3^ Department of Chemistry University of Manchester Manchester UK; ^4^ Manchester Institute of Biotechnology University of Manchester Manchester UK

## Abstract

An enzyme's ability to react with diverse substrates while maintaining high yield and selectivity, termed “substrate promiscuity,” is a highly sought‐after property in biocatalysts. Traditional directed evolution typically relies on single‐substrate screening, which enables adaptive evolutionary pathways but provides limited information on promiscuity. This approach can inadvertently narrow substrate scope, and it is often unclear whether alternative mutational paths might have facilitated broader reactivity. Here, we review substrate multiplexed screening (SUMS), a strategy that places substrates in direct competition and quantifies multiple products simultaneously. When enzymes operate in competitive environments, such as living cells or multi‐enzyme cascades, SUMS directly mirrors the intended use. We discuss how SUMS generates promiscuity information that can guide engineering, even when the final application involves individual substrates. Examples illustrate how SUMS can increase information density relative to parallel screening and, in select cases, reveal activity shifts that are invisible to single‐substrate methods. We discuss the strengths and limitations of SUMS and highlight instances where its application led to enzymes with broad or complementary scopes, identified allosteric mutations, or enabled the circumvention of negative epistasis.

## Graphical abstract

Through historical context and recent examples, this Minireview summarizes the strengths, limitations, and practical considerations for the implementation of substrate multiplexed screening (SUMS) in enzyme evolution.

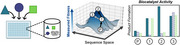

## 1 Introduction

**FIGURE 1:**
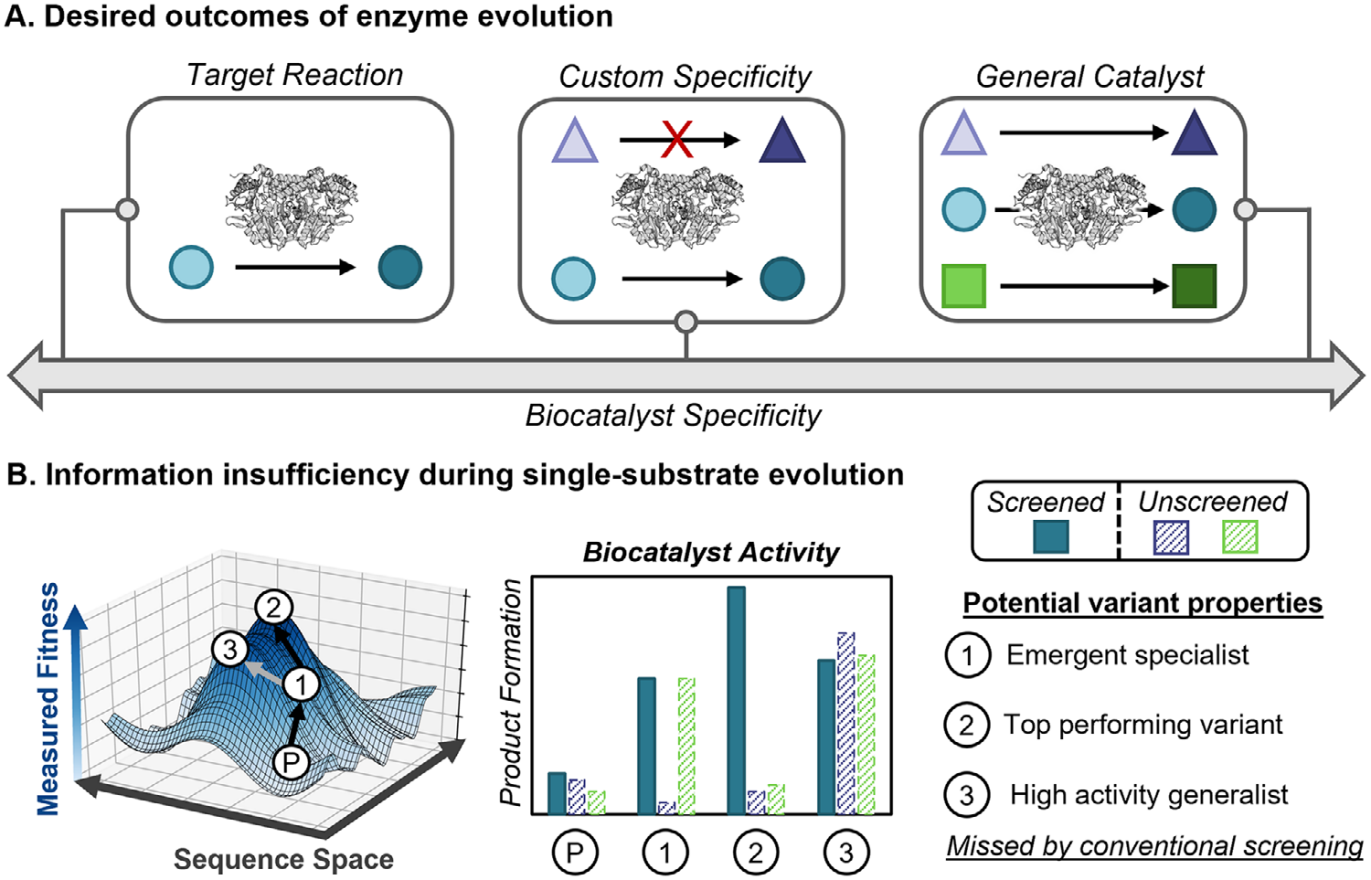
Substrate promiscuity in biocatalysis. (A) Biocatalysts may benefit from a range of specificity profiles depending on their application. (B) Directed evolution reliably increases activity, but changes in activity on related substrates are not captured via single‐substrate screening methods. The parent enzyme is represented with the letter ‘P’ and potential evolutionary steps are enumerated thereafter. Single‐substrate screening would identify variant 2 as the most fit but would not identify the desirable properties latent in variants 1 or 3.

Directed evolution has brought many classes of enzymes into mainstream use for the selective synthesis of complex molecules [1, 2]. Most implementations infer enzyme fitness from activity on a single model substrate [3, 4]. This approach enables adaptive evolutionary pathways across a protein fitness landscape [5, 6, 7], and is exceptionally powerful when the goal is to generate an enzyme that performs a single transformation [2, 8, 9]. However, engineering campaigns often aim to produce one or a set of enzymes that react with many substrates in a well‐defined transformation, a property termed “substrate promiscuity” (Figure 1A) [10, 11]. Single‐substrate screening cannot directly report on promiscuity, and this misalignment can have practical consequences. While directed evolution with a single substrate can yield promiscuous enzymes, intermediates in evolutionary lineages have been reported to have higher activity for alternative substrates of interest than the final variant, implying that the experimental fitness measurement is incomplete [12, 13, 14, 15, 16, 17]. When fitness is measured on a single substrate, alternative mutational pathways that could expand substrate scope go unexplored (Figure 1B). The true frequency of this phenomenon is unknown, as it can only be observed when variants are reassayed against substrate panels. Consequently, laboratory evolution is often repeated to generate sets of enzymes with complementary substrate scopes [16, 18, 19, 20, 21].

Directed evolution is also applied in pursuit of highly specific catalysts, particularly when enzymes operate in complex environments such as living cells or multi‐enzyme cascades. In these settings, enzymes must react with their target substrate while avoiding structurally related off‐target molecules in the reaction milieu [8, 22, 23, 24]. Single‐substrate screening provides no information about whether adaptive mutations also alter selectivity against competing molecules. Even mutations that are “neutral” in a screen can have significant effects on reactivity of substrates that are not under selective pressure [25]. These blind spots can therefore have direct consequences for catalyst performance.

**FIGURE 2:**
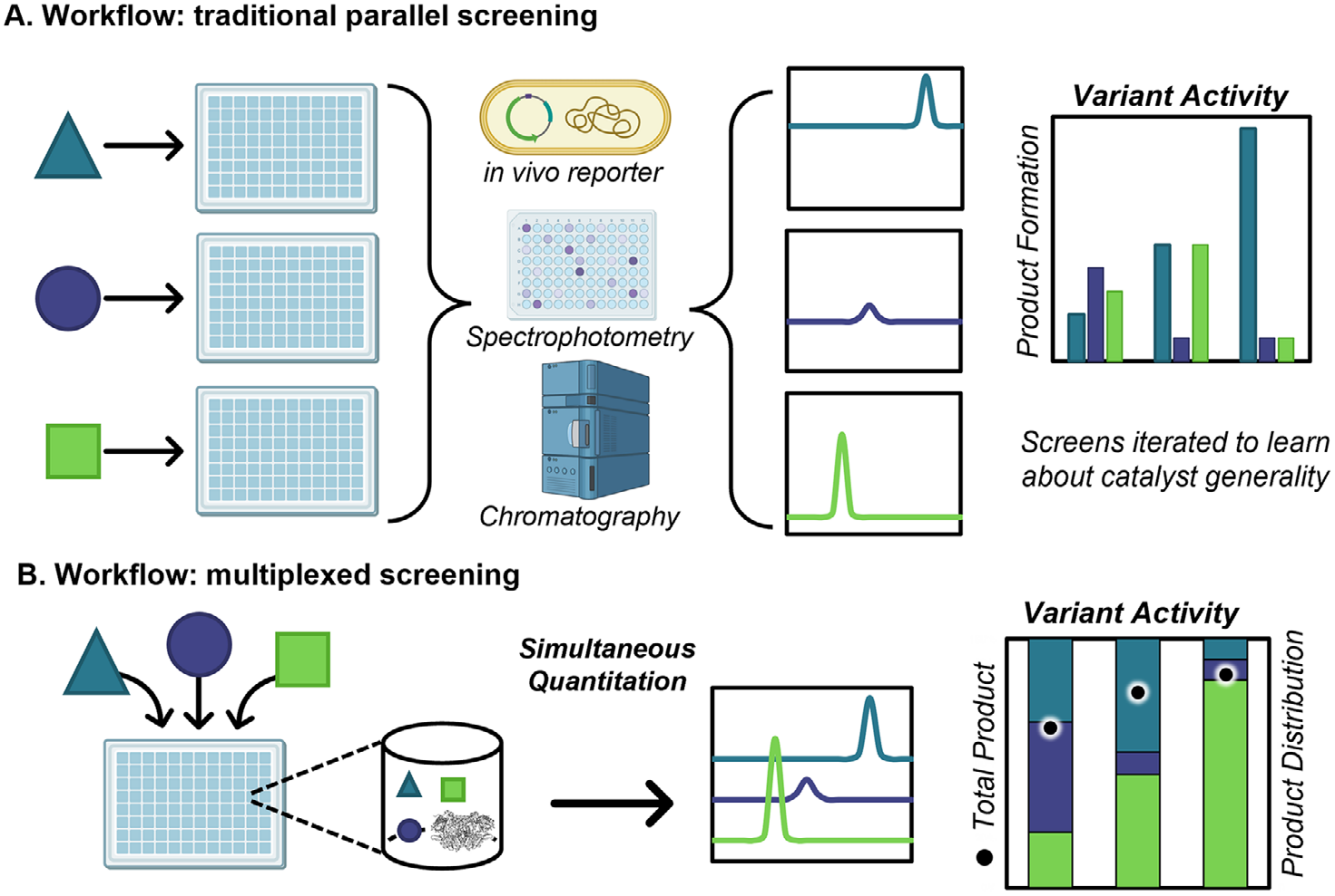
Screening workflows in protein engineering. (A) In traditional approaches, a single substrate is used and many possible selection and screening approaches can be leveraged to rapidly identify desirable variants. Screening reactions must be repeated to gain information on catalyst generality. (B) SUMS places reactants in direct competition and frequently relies on modern mass spectrometry to resolve multiple products simultaneously. The resulting data contains information on both total activity (dots) and enzyme promiscuity(colored bars). Icons used from BioRender.

This minireview examines substrate multiplexed screening (SUMS), a technique in which substrates compete in vitro and multiple products are quantified simultaneously (Figure 2). SUMS addresses both challenges outlined above by generating promiscuity information at the screening stage of a campaign, information that single‐substrate screening cannot provide. We begin with historical precedents in enzymology, discuss practical strengths and limitations of the approach, and demonstrate how promiscuity data can guide engineering along mutational pathways that could otherwise go undetected.

## 2 Historical Context for Incorporation of Promiscuity Information

The limitations of single‐substrate screening have long been recognized, and two distinct strategies have emerged to address them. The first, parallel screening, involves iteratively rescreening libraries against multiple substrates to map enzyme promiscuity [10, 18, 26]. The second draws on a tradition in enzymology of placing substrates in direct competition to quantitatively assess specificity [27, 28]. SUMS has features of both approaches, combining the promiscuity‐mapping goal of parallel screening with the competition format of enzymological assays.

### 2.1 Parallel Screening: Incorporating Promiscuity Information in Directed Evolution

A spectrum of strategies have been used to incorporate substrate promiscuity information during directed evolution campaigns. One common approach is for researchers to use a single substrate for high‐throughput screening and then assess the promiscuity of any hits on an expanded panel of substrates before continuing evolution [29, 30, 31, 32]. Sometimes variants identified this way are evolved further with new substrates [33, 34], as is pursued from the outset during substrate walking [29, 35, 36, 37].

In some cases, researchers iteratively screen libraries on substrates in parallel to capture substrate promiscuity information [38, 39, 40]. The parallel screens may be analyzed independently, or pooled prior to injection on an instrument that can distinguish between multiple products, as is increasingly common in small molecule catalyst development [41, 42, 43]. In an extensive and early example of parallel screening, Reetz et al., used a colorimetric screen to identify eight esterase variants with complementary activity on 11 substrates [18]. When automated liquid handling is available, parallel screening with pooled‐product analysis is particularly powerful and does not require consideration of competition kinetics.

The successes of parallel screening demonstrate how promiscuity information can meaningfully guide directed evolution. However, each additional substrate screened requires an independent reaction setup, which adds procedural complexity and subdivides the finite supply of each variant. Using SUMS, promiscuity information is efficiently generated from a single expression plate and reaction setup (Figure 2B). Additionally, if the end use of the enzyme is a competition setting such as in a cascade or in vivo, parallel screening alone may not provide an accurate picture of catalyst performance.

### 2.2 Competition Screening: Rapid Enzyme Characterization

Application of SUMS to directed evolution has been anticipated by enzymologists, who have long assessed enzyme specificity by screening with substrates in competition [28, 44, 45]. For multi‐enzyme cascades and synthetic biology applications requiring strict specificity, SUMS directly simulates the competitive, in vivo environment. This approach enables selection of variants with desired on‐target activity while excluding those that catalyze off‐target reactions. In biological settings, such specificity is often the primary criteria during evolution [22, 23, 46, 47, 48, 49, 50, 51, 52]. For organic chemistry applications, competition conditions are typically a proxy for single‐substrate activity. The introduction of kinetic complexity, such as substrate inhibition, differential reaction rates, and ion suppression can complicate interpretation of activity profiles. Therefore, this minireview provides straightforward strategies to implement SUMS for biocatalysis and interpret the resulting activity and promiscuity information to enhance laboratory evolution toward a desired substrate scope.

## 3 Fundamentals of Engineering With SUMS

McDonald et al. demonstrated SUMS on two kinetically and structurally distinct enzymes to establish assay design and data interpretation principles for protein engineering contexts [53]. SUMS data naturally support two levels of analysis. At the simplest level, the response of each substrate can be evaluated independently: extracting fold‐changes for individual products and making engineering decisions accordingly, as one would from a conventional single‐substrate screen. This approach captures throughput benefits of multiplexing while remaining analytically familiar. At a second level, the relative distribution of products among competing substrates directly reports on enzyme promiscuity, providing qualitatively different information that cannot be recovered from single‐substrate data. The case studies below illustrate how both analytical modes and the interplay between them can guide engineering decisions.

### 3.1 Identification of Complementary Enzyme Specialists

**FIGURE 3:**
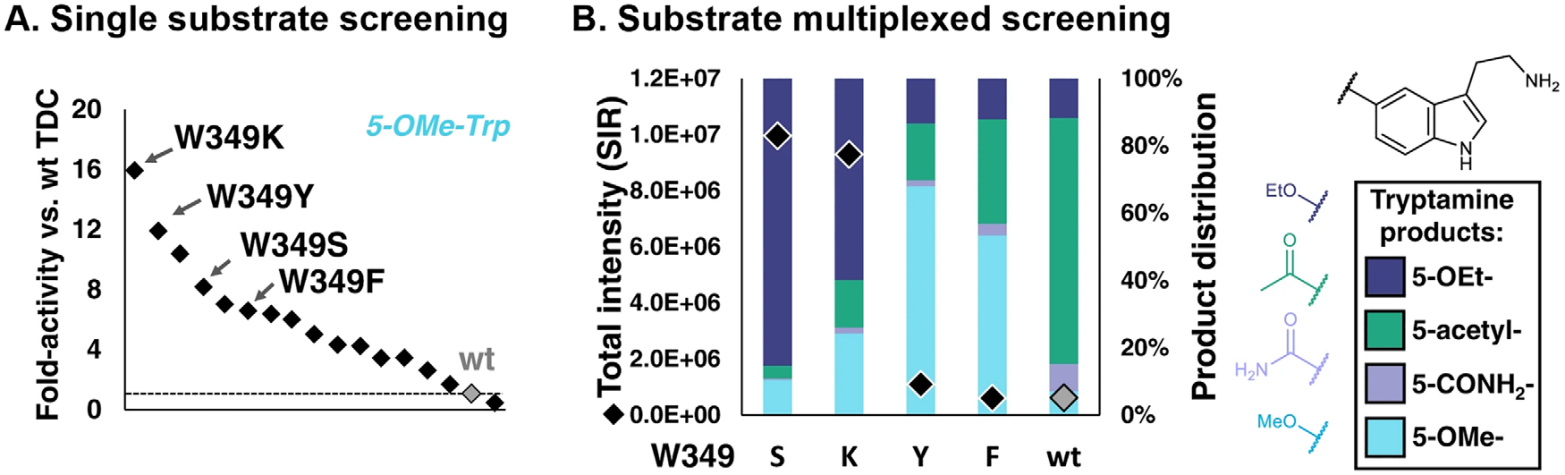
Comparison of screening methods: SSM library at W349 of TDC. (A) The library was screened on 5‐methoxy‐tryptophan. The parent activity is represented by the gray diamond. (B) The library was screened on an equimolar mixture of five‐substituted Trps. The total intensity of all products by MS is depicted by the black diamonds, with the parent activity depicted by the gray diamond. The product distribution is depicted by the colored bars, which correspond to the different tryptamine products. Figure reproduced from McDonald et al. [53].

Tryptophan decarboxylase (TDC) natively catalyzes the unimolecular decarboxylation of l‐tryptophan (Trp) and its analogs into tryptamines [54]. The engineering campaign sought to expand the scope of TDC, with emphasis on five‐substituted Trps. A head‐to‐head comparison of SUMS to single‐substrate screening was performed using site‐saturation mutagenesis (SSM) in the active site at W349 (Figure 3A). The single‐substrate library data and the SUMS library data correlated well, with W349K appearing as a good variant in each case. However, the SUMS library revealed that variants displayed asymmetric increases in activity for the different five‐substituted Trps, highlighting how activity may not be consistent even among closely related substrates (Figure 3B). These results show how multiplexed screening can increase information density without compromising on throughput or iterating rounds of evolution.

**FIGURE 4:**
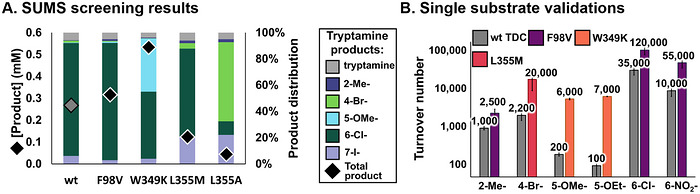
TDC SUMS evolution. (A) Variants of interest identified from SUMS screens. (B) Single‐substrate reactions using the complementary specialist TDCs. Figure reproduced from McDonald et al. [53].

When applying SUMS to differently substituted Trps, MS detection enabled straightforward, simultaneous assessment of promiscuity effects by choosing substrates with distinct masses. Activity increases observed during SUMS screening (Figure 4A) were validated in single‐substrate reactions with purified enzymes (Figure 4B). Notably, variants were often activated for substrates not included in the screening mixture. These results demonstrate that SUMS rapidly identifies beneficial mutations without requiring detailed kinetic analysis.

### 3.2 Navigating Substrate Inhibition Effects

**FIGURE 5:**
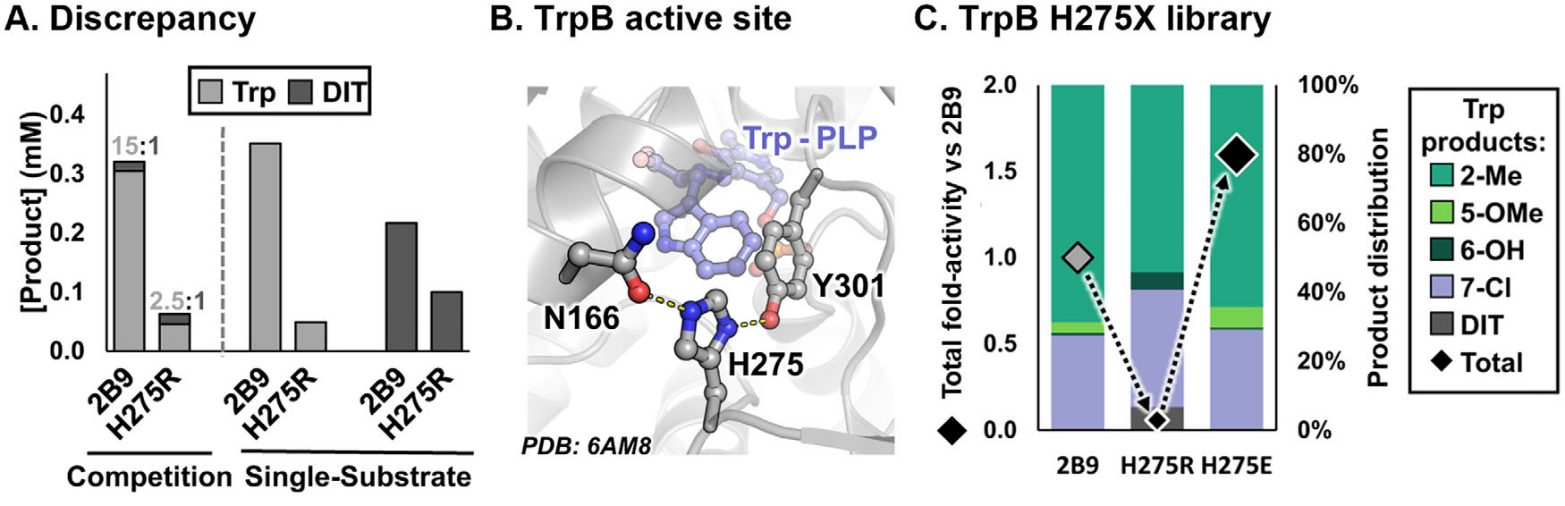
Alleviation of inhibition leads to identification of the activated variant. (A) When compared to parent 2B9, H275R makes more DIT product under competition conditions. Under single‐substrate conditions, however, H275R is less efficient than the parent at producing either Trp or DIT. (B) TrpB active site model (PBD ID: 6AM8) depicting the second‐sphere residue H275 and its possible interactors. (C) Key variants from the SUMS H275X SSM library. Total fold‐activity compared to parent is represented by the black diamonds, with parent activity represented by the gray diamond. Product distribution is depicted by the bars, which correspond to the different Trp products. Figure reproduced from McDonald et al. [53].

Directed evolution of the tryptophan synthase *β*‐subunit, TrpB, revealed how substrate inhibition can complicate SUMS interpretation. TrpB catalyzes a bimolecular reaction, wherein catalytic efficiency is not necessarily correlated to specificity [55]. Starting from a previously engineered, stand‐alone TrpB [12], SUMS of global random mutagenesis libraries revealed generally activating mutations. However, one variant, H275R, demonstrated a discrepancy between SUMS and single‐substrate validation (Figure 5). Under multiplexing conditions, H275R produced more dihydroisotryptophan (DIT) than the parent. Single‐substrate validation revealed lower catalytic efficiency with indolines, an apparent contradiction. The H275R mutation caused a larger decrease in catalytic efficiency with indole than with indoline. Because substrates are inherently competitive inhibitors in SUMS, the mutation's asymmetric effect on catalytic efficiency alleviated inhibition of DIT formation only under competition (Figure 5A). This artifact was not a false positive but reflects differential substrate inhibition. The effect can be minimized by choosing substrates that react to similar degrees, which is empirically determinable without detailed kinetic analysis.

The asymmetric change in activity caused by H275R, a “promiscuity shift”, indicated this second‐sphere residue strongly influences active site chemistry (Figure 5B). SSM at this position identified H275E (Figure 5C) with increased activity on both screened and nonscreened substrates (data not shown) [53]. In this way, a promiscuity shift revealed by SUMS identified a “hotspot” for influencing enzyme activity outside the active site.

These case studies establish practical guidelines for SUMS implementation. Engineering TDC demonstrated how SUMS rapidly identifies complementary enzyme specialists without iterative rescreening. Mutagenesis of TrpB revealed that substrate inhibition can create interpretable artifacts when substrates have dramatically different reactivity. Importantly, neither campaign required knowledge of kinetic parameters or initial velocity conditions. Instead, straightforward analysis of “activity profiles” comprised of total activity and product distribution identified beneficial mutations, including a ‘hotspot’ where SSM led to a generally activated variant.

## 4 Short‐Term SUMS Campaigns: Maintaining and Expanding Scope

### 4.1 Maintaining a Pre‐Existing Promiscuity Profile

Engineering may intend to generally increase the activity of an enzyme that already has a broad scope. The following examples demonstrate SUMS‐guided evolution where the specificity profiles are largely maintained during catalyst optimization.

#### 4.1.1 Multienzyme Cascade: TDC

One‐pot cascade synthesis of 1,2‐amino alcohols presented a need for custom enzyme specificity. The target cascade combined wild‐type L‐threonine transaldolase (ObiH) with RgnTDC. ObiH uses L‐Thr as a substrate to produce *β*‐hydroxy noncanonical amino acids (ncAAs), which TDC then decarboxylates to yield 1,2‐amino alcohols [56, 57]. Native TDC had no activity on L‐Thr, essential to avoid consuming ObiH's substrate, and trace activity on desired *β*‐hydroxy ncAAs. Rather than seek a true generalist, promiscuity profiles from SUMS were used to identify variants that had increased activity on *β*‐hydroxy amino acids but remained inert with L‐Thr. This strategy enabled a one‐pot cascade and demonstrated the utility of SUMS for engineering specificity in multi‐enzyme systems [1, 2].

#### 4.1.2 Maintenance of Generalist Activity: TrpB

**FIGURE 6:**
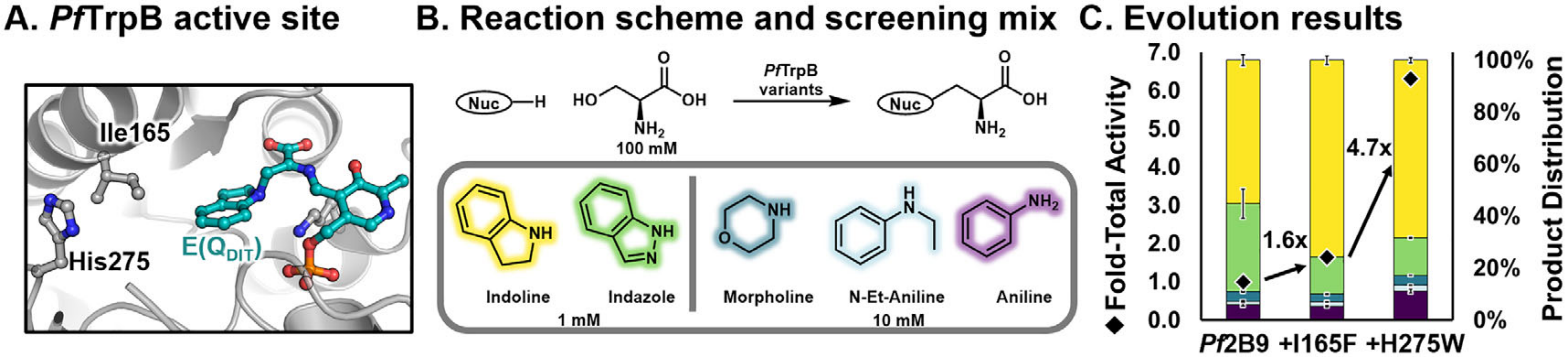
TrpB *N*‐alkylation SUMS evolution. (A) TrpB 2B9 (PDB ID: 6AM8) [59] active site depicting residues that would be mutated in gray superimposed with the product‐bound PLP E(*Q*_DIT_) in cyan (PDB ID: 3CEP) [60]. (B) General reaction scheme of TrpB with the SUMS nucleophile mixture. (C) The activity profiles of the lineage are depicted. The fold activity compared to the parent (*Pf*2B9) is depicted by black diamonds. Product distribution is depicted by colored bars, which correspond to amino acid products. Figure adapted from Villalona et al. [58].

TrpB‐2B9 was observed to have low levels of promiscuous *N*‐alkylation activity with diverse amines. Villalona et al. performed directed evolution to globally increase activity while maintaining the generalist promiscuity profile [58]. Two rounds of SUMS‐based evolution resulted in a variant bearing two additional mutations (Figure 6). Despite both mutations being proximal to the active site, the desirable promiscuity was maintained while increasing activity with all screened nucleophiles. This demonstrates that SUMS enables selection for global activity enhancement without the generalist‐specialist trade‐off that can accompany adaptive evolution.

### 4.2 Expanding Promiscuity

Direct observation of each variant's promiscuity through SUMS enables informed mutation selection. This approach can identify a single generalist catalyst or several complementary catalysts that collectively cover the desired chemical space. Here, we discuss examples of SUMS‐based engineering to expand the scope of biocatalytic transformations.

#### 4.2.1 Active Site Engineering: *Mth*UPO

**FIGURE 7:**
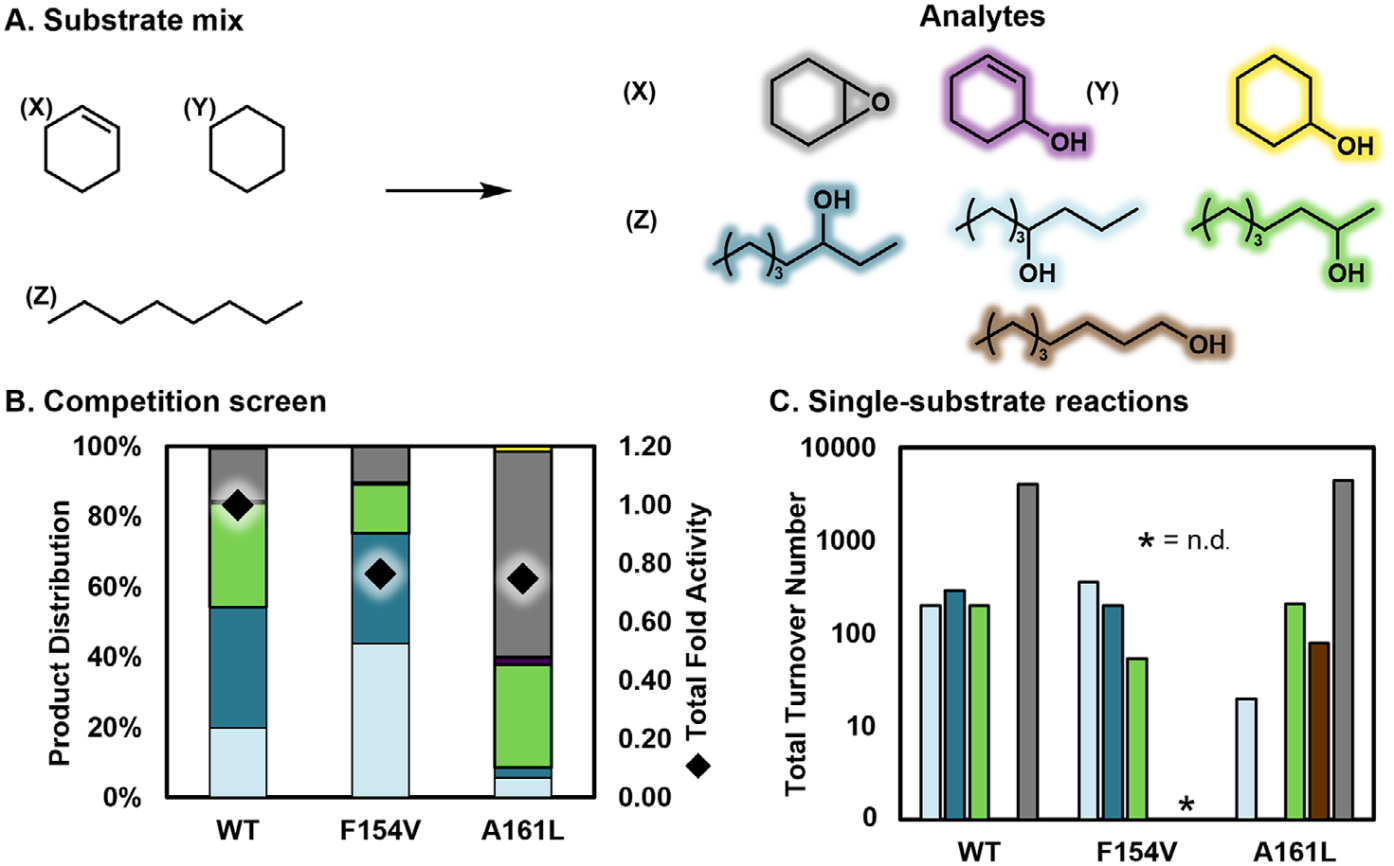
*Mth*UPO SUMS evolution. (A) The substrate mixture and corresponding products were observed. The letters indicate which products are derived from each substrate. (B) Activity profiles for the variants of interest identified during screening. The total fold activity is depicted by black diamonds. The product distribution is depicted by the colored bars. (C) Single‐substrate reactions for F154V and A161L demonstrate the differences in observed activity and regioselectivity of the variants compared to the parent. Hydroxylated products of substrates (X) and (Y) were not evaluated. F154V was not evaluated with cyclohexene (noted with *). Data adapted from Knorrscheidt et al. [61].

Knorrscheidt et al. engineered a peroxygenase from *Myceliophthora thermophila* (*Mth*UPO) to catalyze oxidation of unactivated C*sp^3^*‐H bonds [61]. A single round of active‐site SSM evaluated oxidation of a mixture of octane, cyclohexane, and cyclohexene (Figure 7A). F154V and A161L displayed promiscuity shifts compared to the parent, even though overall activity was lower. Single‐substrate reactions validated a modest improvement in 4‐octanol formation from F154V. Notably, single‐substrate reactions also revealed that A161L was capable of 1‐octanol formation without overoxidation, a challenging outcome not detectable in the initial screening mixture. A161L's distinct promiscuity pattern anticipated its unique selectivity for 1‐octanol under single‐substrate conditions. This example demonstrates that SUMS activity profiles remain predictive even when products are masked during screening.

#### 4.2.2 SUMS‐Guided Library Generation: TDC

**FIGURE 8:**
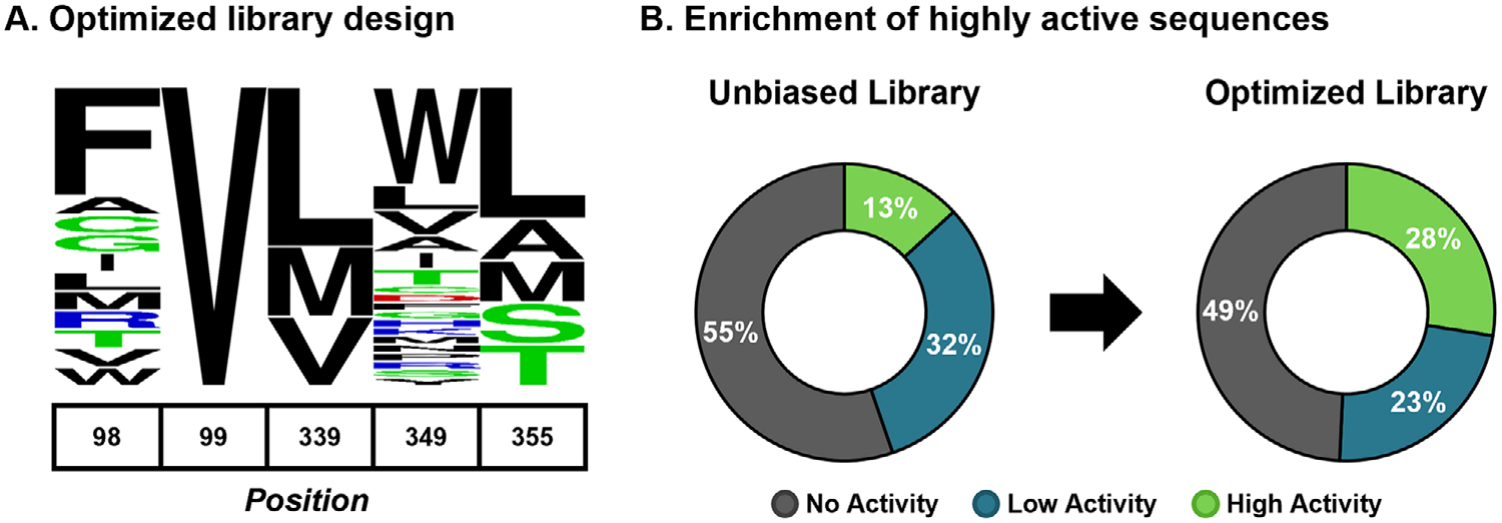
Using SUMS data to optimize library design. (A) WebLogo of the expected mutation distribution in a computationally optimized recombination library at five sites, representing a diverse sequence space. (B) Results of screening an unbiased recombination library versus results of screening the optimized library. “High activity” is defined as >1.5 fold parent activity; “no activity” is defined as < 0.3 fold parent activity. Data adapted from McDonald et al. [62].

McDonald et al. used SUMS to identify sequence‐diverse ncAA decarboxylases [62]. Prior work (Section 3.1) showed active‐site mutations produced idiosyncratic specificity changes, making a single generalist unlikely [53]. Therefore, a panel of diverse ncAA decarboxylases was sought that had activity on pharmacologically relevant four‐ and five‐substituted Trps. A recombination library across five sites was assayed via SUMS (Figure 8). These screens differentiated variants with universally low activity from those with total activity comparable to the parent but divergent specificity, the latter indicating productive sequence space. This sequence‐function data trained a logistic regression model identifying mutation combinations that caused negative epistasis, enabling the design of an optimized library. The second‐generation library doubled the frequency of high‐activity variants while maintaining high mutational diversity, yielding a panel of complementary specialists covering the target substrate scope.

These examples demonstrate how SUMS enables nonadaptive evolution by revealing promiscuity shifts independent of total activity. Rather than iteratively optimizing toward a single model substrate, which risks specialization [15, 63, 64, 65, 66], practitioners can identify complementary variants or computationally optimize libraries to efficiently span desired chemical space in short campaigns.

## 5 Extended SUMS Campaigns: Navigating Complex Sequence Space

SUMS campaigns can also leverage promiscuity information to navigate complex sequence space inaccessible to adaptive evolution. The following examples demonstrate multi‐generational campaigns that include steps in which promiscuity shifts, rather than activity increases, guided mutation selection. This strategy identified distal allosteric residues and circumvented negative epistasis, enabling nonintuitive evolutionary trajectories toward highly active, promiscuous catalysts.

### 5.1 Malonyl CoA Synthetase: MatB

The Lynch lab applied SUMS to overcome a major challenge in polyketide engineering: generating nonnatural malonyl‐CoA extender units for polyketide synthases (PKS) [67, 68]. Malonyl‐CoA synthetase (MatB) exhibits high specificity, and chemical synthesis of CoA analogs is prohibitively lengthy [67]. Using “multi‐agent screening” (synonymous with SUMS), MatB promiscuity was dramatically expanded with non‐natural malonate derivatives [69]. Two protein regions flanking the active site, including positions known to affect specificity, were subjected to SSM. Libraries were screened by testing pooled variants (∼25 per pool) against a mixture of 18 malonate analogs, enabling rapid deconvolution of active variants from inactive ones. Remarkably, beneficial mutations averaged 18.2 Å from the active site, well beyond typical mutagenesis targets. This demonstrates the SUMS utility for identifying distal residues that influence catalysis, a persistent challenge when relying on structural intuition alone.

Recombination of beneficial mutations from SSM yielded 25 variants with expanded promiscuity. Analysis revealed substrate‐dependent epistasis: mutations F189M and P232L each decreased activity individually but synergistically recovered activity when combined. Such examples of negative (or reciprocal) sign epistasis are exceedingly rare in laboratory evolution [70]. Over 30% of top variants contained both mutations, validating their cooperative importance for broad promiscuity. By using promiscuity shifts to justify including activity‐decreasing mutations in recombination libraries, SUMS enabled the discovery and exploitation of epistatic interactions inaccessible to adaptive evolution.

### 5.2 NRPS Adenylation Domain: SrfAC

A SUMS approach, called HAMA, was developed to scan the adenylation domains of non‐ribosomal peptide synthetases (NRPS's), which react with diverse amino acids [47]. After establishing the utility of HAMA for rapid enzyme characterization without iterative single‐substrate assay, Stanišić et al. explored directed evolution of the NRPS SrfAC with HAMA [73]. Starting from a wild‐type enzyme [71], the FuncLib computational approach was applied to design a focused recombination library across three sites [72]. SUMS identified a triple mutant that had expanded promiscuity. Researchers then screened SSM libraries on this generalist enzyme to identify positions that lead to increased specificity. Consistent with bioinformatic approaches, these experiments revealed how small changes to an active site can drive large specificity changes. These data showcase how promiscuity information efficiently extracted under competition conditions provides actionable guidance for laboratory enzyme evolution.

### 5.3 Decarboxylative Aldolase: UstD

Campbell et al. evolved decarboxylative aldolase UstD2.0 to accept ketone electrophiles, a reaction thermodynamically unfavorable for most aldolases [73, 74, 75, 76]. UstD thus presented an authentically challenging engineering target: minimal parent activity toward ketones, a non‐thermostable scaffold, and a transformation that must avoid shunt reactions with unfavorable thermodynamics. SUMS enabled a multi‐round campaign in which promiscuity shifts from low‐activity variants revealed distal allosteric hotspots, demonstrating how the approach navigates genuinely difficult engineering problems by leveraging non‐adaptive evolutionary steps.

**FIGURE 9:**
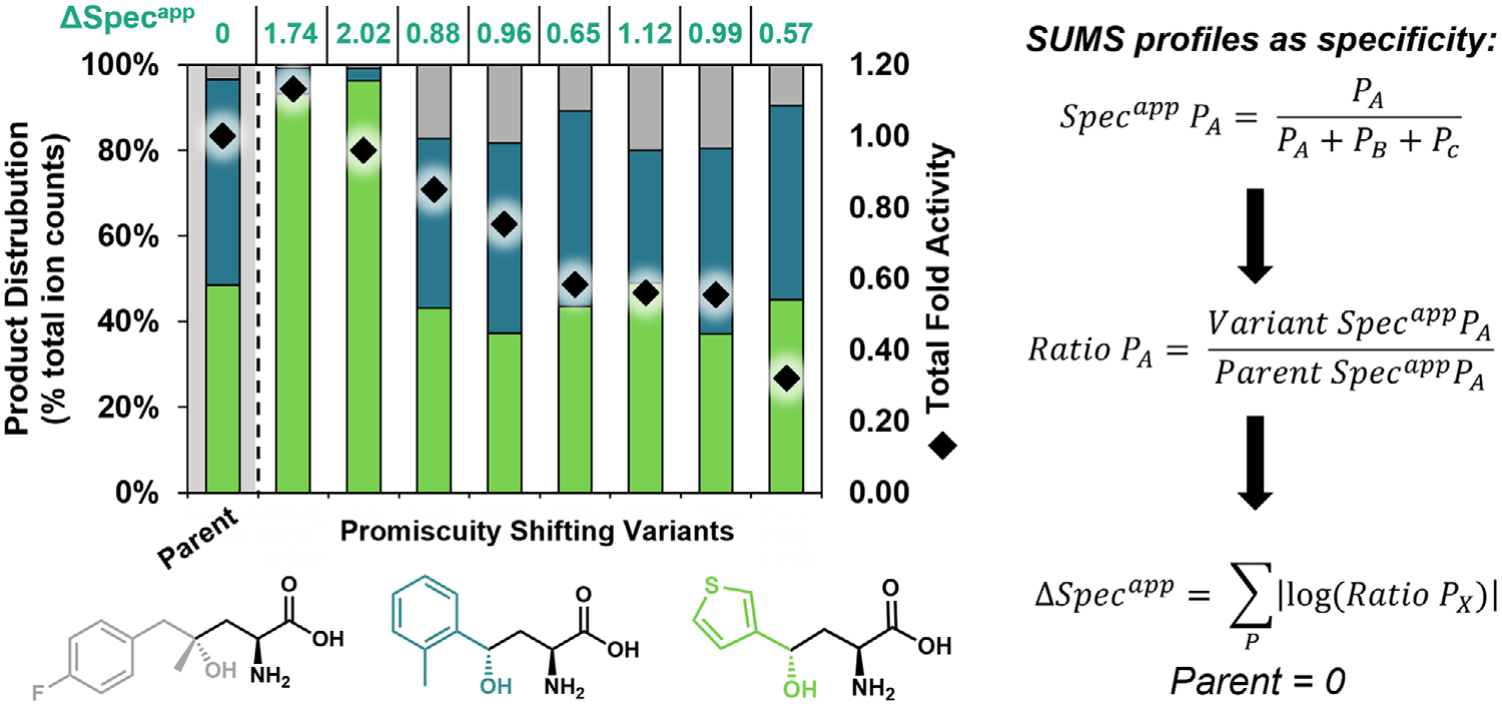
SUMS results from global random mutagenesis. The total activity of all products is depicted by the black dots. The product distribution is depicted by the colored bars that correspond to the different amino acid products. The calculated change in specificity compared to the parent (Δ*Spec* ^app^) is shown above the chart. A Δ*Spec* ^app^ of 0.5 is considered a significant shift in promiscuity. The formulas used to calculate Δ*Spec* ^app^ are shown on the right, with P representing product abundancies for substrates A, B, and C. Data adapted from Campbell et al. [75].

An initial round of global random mutagenesis screened variants on a substrate mixture containing a ketone and two aldehydes. No variants showed universal activity increases, but some displayed shifts in promiscuity despite reduced total activity (Figure 9). This distinction is critical: *variants with reduced activity but unchanged promiscuity suggest decreased protein stability or expression*, offering no evolutionary insight. In contrast, variants with reduced activity and shifted promiscuity indicate altered active‐site function: the mutations influence catalysis in substrate‐specific ways. To quantify these shifts independent of total activity, Campbell et al. calculated each enzyme's apparent specificity (*Spec* ^app^) for each of the possible products. Appropriate treatment of fold‐activity changes relative to the parent led to a simple parameter, Δ*Spec* ^app^ (Figure 9), that captures changes in enzyme promiscuity independent of changes to overall activity. Variants with Δ*Spec* ^app^ > 0.5 were identified as worthy of exploring via SSM.

This quantitative metric revealed eight variants with shifts in promiscuity, but no global increase in activity. SSM was carried out at these putative “hotspots” and revealed many activating mutations, which were incorporated over three short rounds, including recombination. The resulting quadruple variant, UstD^AARIQ^, was comprised entirely of distal mutations at sites first identified by a shift in promiscuity. A final round of active‐site recombination yielded two variants with complementary, broad reactivity toward both ketones and aldehydes. This multi‐round campaign demonstrates how SUMS transforms promiscuity shifts into actionable sequence information, enabling the discovery of distal allosteric networks that traditional activity‐based screening cannot access.

## 6 Conclusions and Outlook

As biocatalysis matures, the complexity of chemical challenges steadily increases. Protein engineers require a toolbox of strategies to efficiently meet diverse catalysis goals. This review has highlighted how multiplexed screening can empower protein engineering. To facilitate wider adoption, we summarize key concepts, design parameters, and benefits of SUMS:

1. Changes in promiscuity are not new in directed evolution. However, researchers have historically been blind to these effects when relying on single‐substrate screens.
2. SUMS can reveal promiscuity shifts with minimal added screening time in the low‐ and medium‐throughput workflows now standard in biocatalysis. No knowledge of underlying kinetics is required.
3. Promiscuity data can distinguish catalytically impaired variants from those with altered substrate recognition, enabling identification of productive sequence space.
4. Engineering may result in either single generalists or complementary specialist panels without reiterating screening.
5. SUMS effectively splits the product signal. If the catalyst has too low activity, it may not be suitable for SUMS until evolution with a single substrate boosts activity.
6. Competitive inhibition between substrates can impede interpretation when substrates have vastly different reactivities. The substrate mix can be empirically tuned prior to evolution so that all products are observed, either by varying substrate identity or concentration.
7. Product inhibition lowers the total signal observed in screens. Because such inhibition lowers the concentration of active enzyme, it has no impact on promiscuity profiles.
8. Promiscuity shifts can identify distal residues influencing catalysis without requiring structural, mechanistic, or dynamic information.
9. Including promiscuity‐shifting mutations in recombination libraries can harness high‐order epistatic interactions, including negative‐sign epistasis, inaccessible to adaptive evolution.

We hypothesize that SUMS will not just provide practical benefits but reinvigorate the fundamental study of enzyme evolution through a multi‐dimensional lens. The studies described here focus on both the substrate space and the sequence space during directed evolution. It has long been acknowledged that substrate choice shapes fitness landscapes [77] and that non‐adaptive steps in evolution provide access to new fitness peaks [78, 79]. SUMS bridges this theoretical foundation with practical biocatalysis, extending these concepts beyond model systems. Further, SUMS generates information‐rich datasets that are compatible with advances in machine learning and artificial intelligence. As biocatalysis increasingly targets transformations distant from natural activities, promiscuity‐guided evolution represents a useful tool in the arsenal of directed evolution practitioners.

## Conflicts of Interest

The authors declare no conflicts of interest.

## Data Availability

Data sharing is not applicable to this article as no new data were created or analyzed in this study.
